# The effect of posterior pericardiotomy after thoracic aortic surgery

**DOI:** 10.1186/s13019-022-01967-8

**Published:** 2022-08-28

**Authors:** Yasumi Maze, Toshiya Tokui, Masahiko Murakami, Daisuke Yamaguchi, Ryosai Inoue, Koji Hirano, Bun Nakamura, Hisato Ito

**Affiliations:** 1 Department of Thoracic and Cardiovascular Surgery, Ise Red Cross Hospital, 1-471-2 Funae, Ise, Mie 516-8512 Japan; 2 Department of Thoracic and Cardiovascular Surgery, Mie University Hospital, Tsu, Mie Japan

**Keywords:** Postoperative atrial fibrillation, Posterior pericardiotomy, Thoracic aortic surgery

## Abstract

**Background:**

Postoperative pericardial effusion is common after cardiovascular surgery. We aimed to examine the effectiveness of posterior pericardiotomy in reducing the incidence of postoperative pericardial effusion and postoperative atrial fibrillation after thoracic aortic surgery.

**Methods:**

This study included 201 patients who underwent thoracic aortic open surgery between January 2014 and November 2021. We compared surgical outcomes between patients who underwent posterior pericardiotomy and those who did not.

**Results:**

The group that did not undergo posterior pericardiotomy had significantly longer mechanical ventilation duration than the group who did, both in the overall (*p* = 0.005) and in the propensity-matched cohorts (*p* = 0.001). The lengths of intensive care unit and hospital stays were significantly longer in the group that did not undergo posterior pericardiotomy compared to the group that did, both in the overall and in the propensity-matched cohorts. The occurrence of postoperative atrial fibrillation and stroke did not differ significantly between the two groups. The amount of pericardial drainage was not significantly lower in the group that underwent posterior pericardiotomy in the overall cohorts (*p* = 0.09), but the difference was significant in the propensity-matched cohorts (*p* = 0.04). The amount of mediastinal drainage was significantly lower in the group that underwent posterior pericardiotomy, both in the overall cohorts (< 0.001) and in the propensity-matched cohort (< 0.001). Late pericardial tamponade occurred significantly more frequently in the group that did not undergo posterior pericardiotomy than in the group that did, both in the overall (*p* = 0.03) and in the propensity-matched cohorts (*p* = 0.03).

**Conclusions:**

Posterior pericardiotomy has no effect on reducing postoperative atrial fibrillation after thoracic aortic surgery. However, posterior pericardiotomy reduced the occurrence of late pericardial tamponade, length of ICU stay, length of hospital stay, and mechanical ventilation duration after surgery.

## Background

Postoperative pericardial effusion is common after cardiovascular surgery [1, 2] and is a risk factor associated with the occurrence of postoperative atrial fibrillation (POAF) [3]. Therefore, reducing postoperative pericardial effusion contributes to the reduction of postoperative complications and cost [4–6]. Since Mulay et al. [7] reported that posterior pericardiotomy (PP) was a simple, safe, and effective method for reducing the incidence of pericardial effusion and thereby the incidence of postoperative supra-ventricular arrhythmias, some studies have reported that PP or posterior pericardial drainage reduces POAF [8–12]. However, although there have been cohort studies examining the effectiveness of pericardial drainage or PP after coronary artery bypass grafting (CABG) or aortic valve replacement (AVR), or after CABG, AVR, and thoracic aortic surgery, these effects have not been evaluated after thoracic aortic surgery alone. In our institution, we have been performing PP in thoracic aortic surgery since 2018. Therefore,the purpose of this study was to examine the effectiveness of PP in thoracic aortic surgery.

## Patients and methods

### Patients

From January 2014 to November 2021, 332 patients underwent open thoracic aortic surgery at our institution. The following patients were excluded from the study population:

- Patients who did not undergo selective cerebral perfusion.
- Patients with previous atrial fibrillation (AF) or paroxysmal atrial fibrillation and a history of ablation treatment
- Patients receiving anti-arrhythmic drugs preoperatively.
- Patients with hyperthyroidism or chronic obstructive pulmonary disease.
- Patients undergoing combined cardiac procedures with valves and/or CABG.
- Patients undergoing redo procedures.
- Patients receiving intra-aortic balloon pumping and/or extracorporeal membrane oxygenation support
- Patients requiring resuscitation with preoperative cardiopulmonary arrest.
- Cases of hospital death and re-exploration for bleeding.
- Patients receiving mechanical ventilation management for 72 h or more after surgery.
- Patients with postoperative reintubation.
- Patients with postoperative Modified Rankin Scale [13] grade 5.

After excluding the 131 patients who met these exclusion criteria, a total of 201 patients were included.

### Ethical statement

All surgical and clinical data collection were performed at Ise Red Cross Hospital, Ise, Japan. Clinical outcome data were obtained from the hospital’s patient records.

This study was approved by the Institutional Review Board of Ise Red Cross Hospital (11/2/2021, approval number ER2021-52), and the need for informed consent was waived due to the retrospective nature of the study. All methods were performed in accordance with the relevant guidelines and regulations.

### Study design

The patients were divided into two groups: those in the PP group underwent posterior pericardiotomy while those in the non-PP group did not. We compared the preoperative characteristics, operative data, and postoperative outcomes between the two groups.

### Operative techniques

Anesthetic medication and surgical techniques were similar in both groups. The operation was performed using median sternotomy in all patients. A regular open distal anastomosis was performed under moderate hypothermic circulatory arrest (25℃) and antegrade selective cerebral perfusion. Before cardiopulmonary bypass (CPB) withdrawal, a longitudinal 5 cm incision was made parallel and posterior to the left phrenic nerve [9] in order to ensure better drainage of the pericardium into the left pleural cavity. However, patients who could not tolerate cardiac abduction or had dense adhesion of the left lung did not undergo posterior pericardiotomy (non-PP group). We have been performing PP since 2018, and patients who underwent surgery before 2018 were included in the non-PP group. Spiral drains were inserted into the pericardium (retrocardiac) and anterior mediastinum in all patients. If the mediastinal pleura was opened by an intraoperative procedure and the pleural cavity was opened, a chest tube was inserted into the pleural cavity regardless of the presence of PP. The pericardium was closed anteriorly as much as possible, and routine chest closure was performed.

A single-branch prosthesis (J Shield Neo; Japan Life Line, Tokyo) was used in ascending aortic or hemiarch replacement, a 4-branch prosthesis (J Shield Neo; Japan Life Line, Tokyo) was used in total arch replacement, and frozen elephant trunk (FET) prosthesis (J graft Frozenix®, Japan Life Line, Tokyo) was used in FET technique.

### Postoperative management

Continuous suction (20 cmH_2_O) was applied to the drains in the intensive care unit (ICU). The drains were milked and stripped at 30 min intervals to ensure tube patency. The pericardial and mediastinal drains were removed when the drainage volume was 100 mL or less in 12 h. Chest tubes were removed when the drainage volume was 150 mL or less in 24 h.

Continuous electrocardiograms (ECGs) of patients were monitored during the first seven postoperative days. If any arrhythmia was suspected or the patient complained of palpitations, ECG monitoring was continued.

AF was defined by the following findings: (1) “absolutely” irregular R-R intervals (in the absence of complete atrioventricular block); (2) no distinct P waves on the surface ECG; and (3) an atrial cycle length (when visible) that is usually less than 200 ms [14]. Patients were considered to have POAF when an episode of AF persisted for longer than 30 s [14].

No prophylactic antiarrhythmic drugs were administered to any of the patients postoperatively. After the diagnosis of AF, if medical treatment was required, administration of agents such as beta-blockers, calcium-channel blockers, procainamide hydrochloride, and amiodarone was considered. If AF caused hemodynamic instability, cardioversion was also considered. Anticoagulation with direct oral anticoagulant or warfarin potassium was considered if AF lasted for more than 48 h.

Two-dimensional echocardiography was performed between the 5th and 14th postoperative day to assess the presence of pericardial effusion. In addition, computed tomography was performed between the 7th and 10th postoperative day to assess the condition of the aorta. On the basis of the image data obtained in these examinations, patients who required pericardial drainage were diagnosed as showing cardiac tamponade. Most patients with cardiac tamponade demonstrated pericardial effusion of 2 cm or more on echocardiography. Additionally, cardiac tamponade presenting after more than seven postoperative days was diagnosed as ‘late pericardial tamponade.’ We defined drainage of pleural effusion or chest tube insertion after removal of the intraoperatively inserted chest tube as ‘late pleural drainage.’ The white blood cell (WBC) count and C-reactive protein (CRP) level were calculated from blood samples collected 5 to 7 days after surgery.

Postoperative management was performed by cardiovascular surgeons, nine cardiovascular surgeons were involved in this study.

### Statistical analysis

All statistical analyses were performed using the statistical software EZR (Easy R) on R commander [15]. Continuous variables are expressed as mean ± standard deviation and were compared using Student’s *t*-test, whereas categorical variables are expressed as counts and percentages and were compared using the *χ*^2^ test. A 1:1 propensity score matching was performed to adjust for baseline differences and reduce confounding variables. Propensity scores were calculated using the following preoperative and operative variables: age, sex, hypertension, creatinine (Cr) level, estimated glomerular rate (eGFR), angiotensin-converting enzyme inhibitor/angiotensin receptor blocker (ACEI/ARB), beta-blocker, Ca-blocker, Japan score, Euro score, operation duration, CPB time, selective cerebral perfusion time, circulatory arrest time, cardiac arrest time, blood loss, and transfusion. After propensity score matching, a matched cohort of 79 patients per group was created. For all analyses, the statistical significance was set at *p* < 0.05.

## Results

### Preoperative characteristics and operative data

The baseline characteristics before matching are summarized in Table 1. The incidence of preoperative beta-blocker oral administration was significantly greater in the non-PP group (33.6% vs. 15.9%, *p* = 0.007). The non-PP group included significantly more cases of dissection type B (12.3% vs 3.4%, *p* = 0.04). Also, the non-PP group included significantly more cases of elective surgery (52.2% vs. 31.8%, *p* = 0.005), whereas the PP-group included significantly more cases of urgent surgery (7.9% vs 18.1%, *p* = 0.04). All preoperative characteristics and operative data with significant intergroup differences were homogenized after propensity score matching (Table 2).

**Table 1:** Baseline characteristics (before matching)

	Non-PP group (*n* = 113)	PP group (*n* = 88)	*p* value
Age	67.6 ± 10.8	69.1 ± 12.6	0.37
Male sex	66(58.4)	45(51.1)	0.37
Body mass index	23.3 ± 3.8	23.9 ± 4.7	0.38
Hypertension	83(73.4)	62(70.4)	0.75
Hyperlipidemia	31(27.4)	25(28.4)	1.0
Diabetes mellitus	6(5.3)	7(7.9)	0.64
CAD	5(4.4)	3(3.4)	1.0
Cr (mg/dL)	0.98 ± 0.93	0.91 ± 0.36	0.47
eGFR (mL/min./1.73m^2^)	64.9 ± 21.3	63.1 ± 20.6	0.53
Hemodialysis	2(1.7)	0	0.59
LVEF(%)	68.8 ± 4.9	68.0 ± 7.0	0.45
LAD(mm)	36.2 ± 6.4	36.5 ± 7.9	0.81
Medication			
ACEI/ARB	56(49.5)	35(39.7)	0.21
beta-blocker	38(33.6)	14(15.9)	0.007
Ca blocker	55(48.6)	35(39.7)	0.26
Etiology			
Dissection type A	79(69.9)	66(75.0)	0.52
Dissection type B	14(12.3)	3(3.4)	0.04
True aneurysm	20(17.6)	19(21.5)	0.60
Elective	59(52.2)	28(31.8)	0.005
Urgent	9(7.9)	16(18.1)	0.04
Emergent	45(39.8)	44(50.0)	0.19
Japan Score			
30 days operative mortality	6.35 ± 5.46	6.01 ± 4.25	0.63
Euro score	5.71 ± 5.10	6.71 ± 6.21	0.21
Procedure			
Ascending replacement	48(42.4)	45(51.1)	0.28
TAR	20(17.6)	14(15.9)	0.88
TAR + FET	44(38.9)	28(31.8)	0.37
Other	1(0.8)	1(1.1)	1.0
Operative data			
Duration(minutes)			
Operation	395.9 ± 81.8	389.2 ± 82.4	0.56
Cardiopulmonary bypass	232.6 ± 46.4	222.8 ± 49.9	0.15
Selective cerebral perfusion	115.5 ± 57.3	111.5 ± 65.5	0.64
Circulatory arrest	45.4 ± 17.9	45.6 ± 14.2	0.91
Cardiac arrest	149.8 ± 27.2	148.5 ± 33.8	0.76
Blood loss (ml)	1641.6 ± 922.1	1808.1 ± 1116.0	0.24
Transfusion(ml)	2186.5 ± 940.2	2292.8 ± 1080.5	0.45
Minimum pharyngeal temp.(℃)	24.5 ± 0.8	24.4 ± 0.5	0.17

*PP* Posterior pericardiotomy, *CAD* Coronary artery disease, *LVEF* Left ventricular ejection fraction,*LAD* Left atrial dimension, *ACEI* Angiotensin converting enzyme inhibitor, *ARB* Angiotensin receptor blocker

*TAR* Total arch replacement, *FET* Frozen elephant trunk

**Table 2:** Baseline characteristics (after matching)

	non-PP group (*n* = 79)	PP group (*n* = 79)	*p* value
Age	68.1 ± 10.2	69.1 ± 12.8	0.59
Male sex	44(55.6))	41(51.8)	0.74
Body mass index	23.1 ± 3.4	23.8 ± 4.8	0.30
Hypertension	53(67.0)	56(70.8)	0.73
Hyperlipidemia	22(27.8)	22(27.8)	1.0
Diabetes mellitus	6(7.5)	7(8.8)	1.0
CAD	4(5.0)	3(3.7)	1.0
Cr (mg/mL)	1.0 ± 1.0	0.9 ± 0.3	0.37
eGFR (mL/min./1.73m^2^)	62.8 ± 21.0	64.0 ± 21.2	0.71
Hemodialysis	1(1.2)	0	1.0
LVEF (%)	69.1 ± 5.1	67.6 ± 7.0	0.26
LAD (mm)	36.2 ± 6.6	37.0 ± 6.9	0.63
Medication			
ACEI/ARB	33(41.7)	32(40.5)	1.0
beta-blocker	13(16.4)	13(16.4)	1.0
Ca blocker	30(37.9)	32(40.5)	0.87
Etiology			
Dissection type A	57(72.1)	60(75.9)	0.71
Dissection type B	4(5.0)	3(3.7)	1.0
True aneurysm	18(22.7)	16(20.2)	0.84
Elective	28(35.4)	25(31.6)	0.73
Urgent	9(11.3)	12(15.1)	0.63
Emergent	42(53.1)	42(53.1)	1.0
Japan Score			
30 days operative mortality	6.7 ± 5.5	6.1 ± 4.2	0.47
Euro score	6.8 ± 5.5	6.6 ± 6.1	0.82
Procedure			
Ascending replacement	36(45.5)	39(49.3)	0.75
TAR	19(24.0)	13(16.4)	0.32
TAR + FET	23(29.1)	26(32.9)	0.73
Other	1(1.2)	1(1.2)	1.0
Operative data			
Duration(minutes)			
Operation	390.8 ± 85.6	388.5 ± 81.7	0.86
Cardiopulmonary bypass	227.8 ± 47.0	222.4 ± 50.6	0.48
Selective cerebral perfusion	109.2 ± 54.8	111.7 ± 62.8	0.78
Circulatory arrest	48.2 ± 17.8	45.7 ± 14.3	0.34
Cardiac arrest	148.5 ± 29.8	148.1 ± 34.3	0.93
Blood loss (ml)	1625.6 ± 940.6	1834.3 ± 1107.6	0.20
Transfusion(ml)	2259.4 ± 952.3	2347.3 ± 1074.7	0.58
Minimum pharyngeal temp.(℃)	24.4 ± 0.7	24.4 ± 0.5	0.57

*PP* Posterior pericardiotomy, *CAD* Coronary artery disease, *LVEF* Left ventricular ejection fraction,*LAD* Left atrial dimension, *ACEI* Angiotensin converting enzyme inhibitor, *ARB* Angiotensin receptor blocker

*TAR* Total arch replacement, *FET* Frozen elephant trunk

Intraoperative anesthetic drugs are shown in Table 3. There was no difference in the anesthetic

**Table 3:** Intraoperative anesthetic drugs

	Before matching cohort		After matching cohort			
	non-PP group (*n* = 113)	PP group (*n* = 88)	p value	non-PP group (*n* = 79)	PP group (*n* = 79)	*p* value
Anesthetics						
Fentanyl(μg/kg)	4.0 ± 2.6	4.5 ± 2.5	0.17	4.1 ± 2.8	4.5 ± 2.5	0.49
Remifentanil hydrochloride(μg/kg)	49.0 ± 18.2	53.4 ± 18.4	0.11	49.5 ± 19.8	54.2 ± 18.7	016
Thiopental sodium(mg/kg)	15.9 ± 6.0	13.7 ± 6.4	0.02	15.9 ± 6.3	14.6 ± 5.7	0.23
Rocuronium bromide(mg/kg)	2.7 ± 0.8	2.7 ± 0.7	0.71	2.7 ± 0.8	2.6 ± 0.7	0.82
Midazolam(mg)	2.7 ± 1.8	2.2 ± 1.9	0.09	2.4 ± 1.5	2.0 ± 1.7	0.14
Propofol(mg/kg)	7.2 ± 3.2	6.9 ± 3.5	0.52	7.3 ± 3.4	7.2 ± 3.2	0.88

*PP* Posterior pericardiotomy

dugs used during the operation between the non-PP group and PP group.

### Postoperative outcomes

The postoperative outcomes are summarized in Table 4. The non-PP group showed a significantly longer mechanical ventilation duration both in the overall (*p* = 0.005) and in the propensity-matched cohorts (*p* = 0.001). Both the length of ICU stay and the length of hospitalization were significantly longer in the non-PP group, both in the overall and in the propensity-matched cohorts. POAF occurrence did not differ significantly between the PP and non-PP groups (before matching, *p* = 0.38; after matching, *p* = 0.26). There was no significant difference in stroke between the PP and non-PP groups (before matching, *p* = 0.54; after matching, *p* = 0.6). The WBC count and CRP level did not differ between the PP and non-PP groups (WBC: before matching, *p* = 0.59; after matching, *p* = 0.87, CRP: before matching, *p* = 0.75; after matching, *p* = 0.69). The amount of pericardial drainage was not significantly lower in the PP group in the overall cohorts (*p* = 0.09), but the difference was significant in the propensity-matched cohorts (*p* = 0.04). The amount of mediastinal drainage was significantly lower in the PP group, both in the overall cohorts and in the propensity-matched cohorts. The duration of indwelling of the pericardial drain and mediastinal drain was significantly shorter in the PP group, both in the overall and in the propensity-matched cohorts. Late left pleural drainage occurred significantly more frequently in the non-PP group, both in the overall (*p* < 0.001) and in the propensity-matched cohorts (*p* < 0.001). Late pericardial tamponade occurred significantly more frequently in the non-PP group, both in the overall (*p* = 0.03) and in the propensity-matched cohorts (*p* = 0.03). Finally, total hospitalization costs did not differ between the PP and non-PP groups.

**Table 4:** Postoperative outcomes

	Before matching cohort			After matching cohort		
	non-PP group (*n* = 113)	PP group (*n* = 88)	*p* value	non-PP group (*n* = 79)	PP group (*n* = 79)	*p* value
Mechanical ventilation time (hr)	13.3 ± 9.1	10.1 ± 6.3	0.005	14.6 ± 10.3	10.2 ± 6.5	0.001
Length of ICU stay (days)	4.1 ± 2.3	3.0 ± 1.5	< 0.001	4.4 ± 2.5	3.0 ± 1.3	< 0.001
POAF	53(46.9)	35(39.7)	0.38	40(50.6)	32(40.5)	0.26
RRT	3(2.6)	0	0.34	3(3.7)	0	0.24
Modified Rankin Scale	2.8 ± 0.7	2.9 ± 0.7	0.27	2.8 ± 0.7	2.9 ± 0.7	0.45
Length of hospital stay(days)	21.5 ± 14.3	17.5 ± 8.2	0.02	21.7 ± 9.4	18.0 ± 8.4	0.01
Stroke	10(8.8)	11(12.5)	0.54	7(8.8)	10(12.6)	0.60
WBC	8231.8 ± 2729.5	8431.8 ± 2572.7	0.59	8532.9 ± 2915.4	8463.2 ± 2585.3	0.87
CRP	11.0 ± 5.6	11.3 ± 6.7	0.75	11.6 ± 6.1	11.2 ± 6.8	0.69
Parameters of drains (operative)						
Pericardial total drainage(ml)	381.1 ± 361.8	309.4 ± 191.2	0.09	409.5 ± 412.9	303.7 ± 190.1	0.04
Duration of pericardial drain(days)	2.7 ± 1.2	2.1 ± 0.6	< 0.001	2.8 ± 1.4	2.1 ± 0.7	< 0.001
Mediastinal total drainage(ml)	485.0 ± 341.1	330.1 ± 165.9	< 0.001	496.5 ± 331.3	331.9 ± 168.4	< 0.001
Duration of mediastinal drain(days)	2.8 ± 1.3	2.1 ± 0.4	< 0.001	2.8 ± 1.4	2.1 ± 0.5	< 0.001
Left pleural drain n(%)	29(25.6)	88(100)	< 0.001	18(22.7)	79(100)	< 0.001
Left pleural total drainage(ml)	1124.3 ± 833.5	904.4 ± 640.9	0.14	1133.6 ± 977.9	960.6 ± 648.6	0.36
Duration of left pleural drain(days)	5.1 ± 2.2	3.7 ± 1.9	0.003	4.8 ± 2.6	3.9 ± 1.9	0.09
Right pleural drain *n*(%)	36(31.8)	38(43.1)	0.13	25(31.6)	33(41.7)	0.24
Right pleural total drainage(ml)	677.5 ± 538.5	544.2 ± 459.1	0.25	653.0 ± 544.4	546.0 ± 477.7	0.43
Duration of right pleural drain(days)	4.1 ± 1.8	3.0 ± 1.5	0.006	3.9 ± 1.7	3.1 ± 1.6	0.07
Late left pleural drainage *n*(%)	58(51.3)	16(18.1)	< 0.001	43(54.4)	14(17.7)	< 0.001
Late right pleural drainage *n*(%)	23(20.3)	14(15.9)	0.53	16(20.2)	13(16.4)	0.68
Late pericardial tamponade *n*(%)	10(8.8)	1(1.1)	0.03	8(10.1)	1(1.2)	0.03
Total hospitalization costs(yen)	6,510,123.5 ± 1,264,319.0	6,134,096.0 ± 1,264,319.0	0.08	6,355,609.3 ± 1,408,680.9	6,162,882.7 ± 1,280,492.3	0.36

*PP* Posterior pericardiotomy *RRT* Renal replacement therapy, *POAF* Postoperative atrial fibrillation, *WBC* White blood cell, *CRP* C-reactive protein

## Discussion

In our study, PP had no effect on reducing postoperative atrial fibrillation after thoracic aortic surgery, however PP reduced the occurrence of late pericardial tamponade, length of ICU stay, length of hospital stay, and mechanical ventilation duration after surgery.

The incidence of POAF after cardiovascular surgery is reported in 20–40% of cases [4–6, 16–18], but most of these cases were reported post-CABG, with only a few cases reported post-thoracic aortic surgery [6, 18]. Matsuura et al. [6] reported that the incidence of POAF after aortic arch repair was 52.7%. In previous reports, the incidence of POAF after thoracic aortic surgery was higher than that after CABG. In our study, the incidence of POAF was as high as 45.6%. This may be due to the fact that we defined AF as an AF episode of 30 s or longer, which was stricter than the definitions used in other studies. Nishi et al. [19] reported that aortic surgery was associated with an almost threefold increase in the risk of POAF, and the incidence of AF was higher in patients undergoing thoracic surgery than in those undergoing other types of cardiac surgery. The preservation of the aortic fat pad is correlated with a decreased incidence of postoperative atrial arrythmia [20, 21]. Thoracic aortic open surgery results in the removal of the aortic fat pad and this may cause an increase in POAF. Matsuura et al. [6] also reported that POAF after aortic arch repair was associated with prolonged ICU and postoperative hospital stay. In another study, Almassi et al. [22] concluded that POAF after CABG was associated with a higher in-hospital cost of care. Recently, Eikelboom et al. [16] showed that patients who developed POAF after cardiac surgical intervention had an increased risk of death and stroke at 1 year or more after their operation. Therefore, prevention of POAF is a very important issue.

Although the etiology of POAF is not completely understood, various stimuli and triggers such as pre-existing structural changes of the atria related to hypertension, mechanical damage, volume overload, age, intraoperative atrial ischemia, electrolyte imbalance, and pericardial lesions are believed to play a role in its pathogenesis [19, 23, 24]. Greenberg et al. [5] pointed out the interplay between pre-existing physiological components, and local and systemic inflammation as the pathogenesis of POAF. In local inflammation, especially, postoperative pericardial fluid is highly oxidative and contains blood, hemolyzed blood cells, hemoglobin, and high levels of inflammatory markers that causes leukocyte and platelet activation, thus contact between those inflammatory cells and the cardiac tissue likely plays a role in the pathogenesis of POAF [25].

Mulay et al. [7] demonstrated a reduction in both pericardial effusion and related supraventricular arrhythmias with PP. Since then, there have been conflicting reports on the efficacy of PP for POAF [7–12, 22]. PP provides an effective pathway for drainage of pericardial effusion, which has the above-mentioned inflammatory-inducing components, to the pleural cavity. Blood drainage from the pericardial space by maintaining drain patency in the early hours after surgery may reduce the incidence of POAF and other postoperative complications [5, 26]. There is no study on the POAF-reduction effect of PP alone in the thoracic aortic surgery cohort, and we have been performing PP to reduce the incidence of POAF in thoracic aortic surgery since 2018. Nevertheless, in our study, PP had no significant effect on POAF or postoperative stroke reduction. However, it did result in significant reductions in the postoperative mechanical ventilation duration, length of ICU stay, and length of hospital stay. Furthermore, the durations of indwelling of the pericardial and mediastinal drains were significantly shorter in the PP group, and late left pleural drainage occurred significantly more frequently in the non-PP group. Also, the amount of drainage in the pericardial and mediastinal drains was lower in the PP group, and late pericardial tamponade occurred more frequently in the non-PP group. Thus, in our study, PP may bring various benefits by inducing pericardial and mediastinal effusions into the left pleural cavity. In other words, the excretion of inflammatory cells from the pericardium and mediastinum may have had a positive effect. Also, in this study, the incidence of POAF was not lowered by the PP, even though PP could decrease the total amount of the postoperative pericardial and mediastinal drainage.

Notably, the mechanical ventilation duration after surgery was reduced in the PP group. However, whether PP reduces the mechanical ventilation duration after surgery remains unclear. Balzer et al. [27] reported that retention of blood around the heart and lungs was associated with ventilation duration after cardiac surgery. It has also been reported that ventilation hours are reduced by maintaining drain patency and drainage of pericardial effusion and pleural effusion [26]. Based on our findings, the amounts of pericardial and mediastinal drainages were significantly lower in the PP group than in the non-PP group. Therefore, we believe that effective drainage of pericardial and mediastinal effusion, which has retained blood, to the left pleural cavity, contributed to shortening the mechanical ventilation duration. In addition, PP reduced the mechanical ventilation duration after surgery, the duration of indwelling of the pericardial and mediastinal drain, the duration of the bilateral pleural drain, and the late pericardial tamponade. We believe that collectively these parameters contributed to the reduction of the lengths of ICU and hospital stay. In our study, there were no differences in the anesthetic drugs used during the operation between the non-PP group and the PP group (Table 3). Therefore, the intraoperative anesthetic drugs did not affect mechanical ventilation duration after surgery. However, a total of nine cardiovascular surgeons were engaged in postoperative management, which may have affected postoperative mechanical ventilation duration.

In this study, no complications were observed with PP, as pointed out by Yorgancioğlu et al. [28]. Thus, PP is a simple and safe technique.

### Limitations

The present study was limited by its retrospective, single-center design. Furthermore, the small number of cases made it difficult to draw a clear conclusion. For instance, PP showed no significant effects on POAF reduction. To this end, large cohort studies with a large number of cases may provide significant results in this regard.

## Conclusions

PP has no effect on reducing POAF after thoracic aortic surgery. However, PP reduces the occurrence of late pericardial tamponade, length of ICU stay, length of hospital stay, and mechanical ventilation duration after surgery by suppressing local and systemic inflammation. Additional large cohort studies are needed to further clarify the effects of PP.

## Data Availability

The datasets used and/or analyzed during the current study are available from the corresponding author on reasonable request.

## Abbreviations

POAF: Postoperative atrial fibrillation
AF: Atrial fibrillation
CABG: Coronary artery bypass grafting
AVR: Aortic valve replacement
PP: Posterior pericardiotomy
CPB: Cardiopulmonary bypass
FET: Frozen elephant trunk
ICU: Intensive care unit
ECG: Electrocardiogram
WBC: White blood cell
CRP: C-reactive protein
eGFR: Estimated glomerular rate
ACEI: Angiotensin converting enzyme inhibitor
ARB: Angiotensin receptor blocker
LVEF: Left ventricular ejection fraction
LAD: Left atrial dimension
TAR: Total arch replacement
RRT: Renal replacement therapy
